# Bone Mineral Density, Bone Turnover Markers and Fractures in Patients with Systemic Sclerosis: A Case Control Study

**DOI:** 10.1371/journal.pone.0066991

**Published:** 2013-06-20

**Authors:** Marco Atteritano, Stefania Sorbara, Gianluca Bagnato, Giovanni Miceli, Donatella Sangari, Salvatore Morgante, Elisa Visalli, Gianfilippo Bagnato

**Affiliations:** Department of Internal Medicine, University of Messina, Messina, Italy; Keio University School of Medicine, Japan

## Abstract

**Objective:**

The aim of our study was to elucidate the pathophysiology of systemic sclerosis-related osteoporosis and the prevalence of vertebral fragility fracture in postmenopausal women with systemic sclerosis (SSc).

**Methodology:**

Fifty-four postmenopausal women with scleroderma and 54 postmenopausal controls matched for age, BMI, and smoking habits were studied. BMD was measured by dual energy-x-ray absorptiometry at spine and femur, and by ultrasonography at calcaneus The markers of bone turnover included serum osteocalcin and urinary deoxypyridinoline. All subjects had a spine X-ray to ascertain the presence of vertebral fractures.

**Results:**

bone mineral density at lumbar spine (BMD 0.78±0.08 vs 0.88±0.07; p<0,001), femoral neck (BMD: 0.56±0.04 vs 0.72±0.07; p<0,001) and total femur (BMD: 0.57±0.04 vs 0.71±0.06; p<0,001) and ultrasound parameter at calcaneus (SI: 80.10±5.10 vs 94.80±6.10 p<0,001) were significantly lower in scleroderma compared with controls; bone turnover markers and parathyroid hormone level were significantly higher in scleroderma compared with controls, while serum of 25(OH)D3 was significantly lower. In scleroderma group the serum levels of 25(OH)D3 significantly correlated with PTH levels, BMD, stiffness index and bone turnover markers. One or more moderate or severe vertebral fractures were found in 13 patients with scleroderma, wherease in control group only one patient had a mild vertebral fracture.

**Conclusion:**

Our data shows, for the first time, that vertebral fractures are frequent in subjects with scleroderma, and suggest that lower levels of 25(OH)D3 may play a role in the risk of osteoporosis and vertebral fractures.

## Introduction

Systemic sclerosis (SSc) is a chronic disease characterized by increased synthesis and deposition of collagen in skin and connective tissue, vascular alteration and immunological disturbances. Recently was demonstrated that SSc itself is a risk factor for osteoporosis [1]. Several other studies had observed a relationship between low bone density and systemic sclerosis [2]–[4]. Low bone density is associated with a greater risk of fragility fractures. Knowledge about the pathophysiology of SSc-related osteoporosis is limited. Vitamin D levels are directly related to bone mineral density in both gender and in diverse race with a maximum density accomplished with levels of 40 ng/mL or more [5]. Vitamin D insufficiency is recognized as one of the important causal factor for low bone mass, since it has a well-known role in regulating calcium and phosphorus metabolism, bone formation and mineralization. Interest in vitamin D has risen in recent years as shown by the exponential increase of publication, above all concerning its extraosseus action and the immunoregolatiry effects. In fact, vitamin D has also immunomodulatory properties with a potential benefit for autoimmune disease [6]. A high prevalence of vitamin D deficiency has been reported in rheumatologic outpatients [7]–[13], including autoimmune diseases such as systemic lupus erythematosus [8]–[12] and rheumatoid arthritis [9], [10], [13], [14]. It has been documented that vitamin D receptors are present on the surface of antigen presenting cells, natural killer cells as well as B and T lymphocytes [15]–[16], explaining multiple immunomodulating effects on both innate and adaptive immune responses. In recent study, Caramaschi P et al have described that SSc patients with vitamin D deficiency showed more severe disease in comparison with SSc patients with vitamin D insufficiency [17]. Braun-Moscovici Y et al have described secondary hyperparathyroidism related to low 25-hydroxyvitamin D level in Mediterranean SSc patients partially explained by low sun exposure [18]. It is possible that vitamin D represent a factor which play a role on bone loss and fracture risk in scleroderma patients. The aim of our study was to determine the prevalence of osteoporosis and fragility fracture in SSc patient.

## Materials and Methods

### Subjects

The study was approved by the Local Ethics Committee for Medical Research, Messina University Hospital “G.Martino” and carried out in accordance with the Helsinki Declaration. All subjects gave their informed written consent. Consecutive patients with newly diagnosis of systemic sclerosis referred from June 2003 to March 2010 to the Unit of Rheumatology in the Department of Internal Medicine of the University of Messina were evaluated. Inclusion criteria were women 49 to 60 yrs of age and with at least 12 months of menopause at baseline, had not had a menstrual period in the preceding year and had not undergone surgically induced menopause, had a follicle-stimulating hormone (FSH) level >50 IU/liter and a serum 17β-estradiol (E2) level ≤100 pmol/liter. Eligibility criteria required the absence of any other rheumatic disorders, clinical or laboratory abnormalities that suggested cardiovascular, hepatic or renal disorders; coagulopathy, use of oral or transdermal estrogen, progestin, androgen or other steroids; use of biphosphonates, cholesterol-lowering therapy, cardiovascular medications, or other therapies that could influence bone metabolism, in particular, systemic or local corticosteroids for more than 1 month overall. Of the 139 patients evaluated fifty-four with inclusion criteria were included in this cross-sectional study. Disease duration was defined as the time elapsed between the onset of first-disease related symptoms and enrolment. The distinction between limited and diffuse cutaneous SSc was made according to the criteria of LeRoy et al. [19]. SSc activity was assessed according to the preliminary composite index proposed by the European Scleroderma Study Group and disease severity was assessed according to the Medsger’s severity score [20], [21]. Fifty-four healthy postmenopausal women matched for age, body mass index (BMI), menopausal age and smoking habits, addressed to our outpatient clinic from their gynecologist for suspicion of osteoporosis served as the control group. Women in both group were white from South Italy. Data collected before start of treatment were used in this cross-sectional study. Clinical data including smoking status (current, former, never), physical activity, food energy, calcium intake, weight history, including maximum weight, were obtained by interview and weight and height were also determined during physical examination. The nutritional variables were determined using 24 hour recall. All subjects were required to have self reported sun exposure of 1 or more hours for day on 5 or more day per week during at least the preceding summer. None of the subjects in both group were on supplementation with calcium and vitamin D.

### Bone Mineral Density and Bone Ultrasound Parameter

All subjects BMD was measured by DXA (Hologic QDR 4500, Waltham, MA) at the lumbar spine (L1–L4 BMD), femoral neck (FN BMD) and total femur (TF-BMD). The results were expressed as T-score (standard deviation-SD-below the mean of young healthy adults). Osteoporosis by BMD criteria was defined as a lumbar spine or hip BMD T-score of −2,5 SD or less, according to the traditional WHO criteria [22] In the same times all patient was measured ultrasound parameter (QUS) of the non dominant heel(Achilles plus, Lunar, Madison, WI). The Achilles Plus measures the speed of sound (SOS), broadband ultrasound attenuation (BUA), and a clinical index called stiffness. Stiffness is calculated automatically by the software according to the following formula: stiffness = (0.67×BUA+0.28×SOS)−420. Standardized procedures were carried out for patient positioning, data acquisition, and system calibrations for both techniques. Manufacturer’s phantoms were used for system calibrations. The measurements of BMD and QUS were made of the non-dominant hip and heel. The coefficients of variation for lumbar spine BMD was 1.2%, for femoral neck BMD 1.3%, for total femur BMD 1,1% and 1.7% for Stiffness index.

### Vertebral Fracture Assessment

All subjects had a lateral thoracic and lumbar spine X-ray to ascertain the presence of vertebral fractures. Anterior, middle, and posterior heights of vertebral T4 to L4 were measured. According to the Genant classification, a vertebral fracture was defined based on reduction in anterior, middle, and/or posterior height: grade 1, 20–25% reduction; grade 2, 25–40% reduction; and grade 3, >40% reduction [23].

### Bone Turnover Metabolism

A 2-hour fasting morning urine sample was collected at the same time of the day to assess urinary excretion of deoxypyridinoline. Deoxypyridinoline was measured by using high-performance liquid chromatography (Bio-Rad Laboratories, Hercules, California). After an overnight fast, venous blood samples were collected between 8 a.m. and 9 a.m. through a polyethylene catheter inserted in a forearm vein. Serum was separated from the blood corpuscles by centrifugation and kept frozen at −70°C until analysis for bone formation, calcium, intact parathyroid hormone, 25-hydroxyvitamin D3, 17β-estradiol, follicle-stimulating hormone. Osteocalcin was performed using an immunoenzymatic assay (Pantec, Turin, Italy). Serum calcium, serum phosphorus and urinary creatinine were measured by using automated routine procedures. Parathyroid hormone, 25-hydroxyvitamin D3 and follicle-stimulating hormone were measured using high-performance liquid chromatography (Bio-Rad Laboratories). 17β-Estradiol was evaluated using a solid-phase immunoassay (Roche Diagnostics, Monza, Italy). The intra- and inter-assay CV were <10% for both tests.

Statistical analyses were performed using StatSoft software (release 4.5). All values were expressed as mean ± SD and percentage. Comparisons between the two groups were performed by Student’s t-test. Multivariate logistic regression analysis was used to adjust for confounders.The percentage of each variable was compared between groups by Fisher’s exact test. Pearson’s correlation coefficient was calculated to evaluate the correlation between two variables. Values of P<0.05 were considered to be significant.

## Results

**Figure 1:**
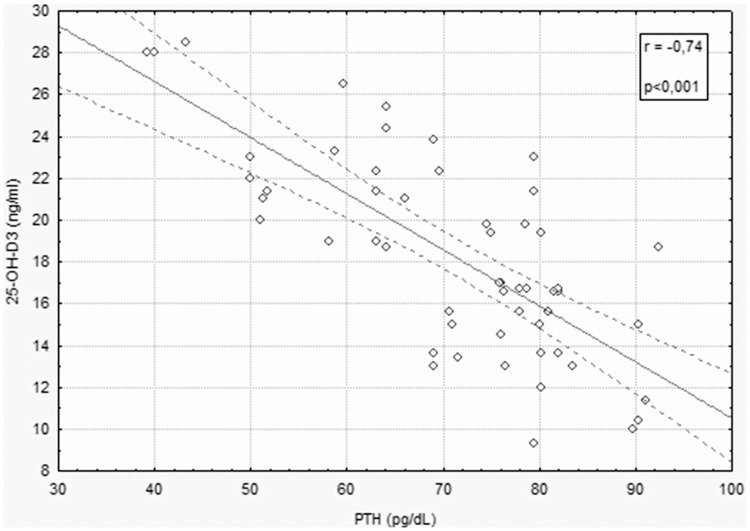
Negative correlation between parathyroid hormone level and 25-hydroxivitamin D level in SSc.

The main demographic and clinical features of participants are given in Table 1. As expected by the matching design of the study, chronological age (p = 0.61), menopausal age (p = 0.43), BMI (p = 0.26) and family history of osteoporosis and fracture (p = 0.45) were comparable in SSc and controls. Other relevant factors associated with bone mass were also alike in both groups (Table 1). Osteodensitometry results and laboratory findings are given in Table 2. The results show that women with systemic sclerosis have a statistically significant lower BMD and T-score, measured at lumbar spine (BMD: 0.78±0.08 g/cm^2^ vs 0.88±0.07 g/cm^2^ p<0.001; T-score: −2.20±0.30 g/cm^2^ vs. −1.20±0.30 g/cm^2^, p<0.001), femoral neck (BMD: 0.56±0.04 g/cm^2^ vs. 0.72±0.07 g/cm^2^, p<0.001; T-score: - 2.60±0.20 g/cm^2^ vs. −1.30±0.30 g/cm^2^, p<0.001) and total femur (BMD: 0.57±0.04 g/cm^2^ vs. 0.71±0.06 g/cm^2^, p<0.001; T-score: −2.50±0.30 g/cm^2^ vs. −1.40±0.30 g/cm^2^, p<0,001). In according to the WHO classification, osteoporosis was found in 12 patients with scleroderma (22%), wherease in control group was found in 4 subject (8%); if we consider also the osteopenia the percentage of low bone mineral density increase in both groups, 68% in SSc and 38% in control group. The ultrasound parameter at calcaneus, Stiffness Index, was significantly lower in SSc in comparison with control subjects (SI: 80.10±5.10% vs. 94.80±6.10%, p<0.001). After adjustment of results for age and potential risk factors for osteoporosis the difference in BMD and QUS parameter between group remained significant. Serum levels of calcium and phosphorus did not differ significantly between the study groups (Table 2). Level of PTH was significantly higher in patients than in control subjects (72.26±11.23 pg/dL vs 39.64±11.90 pg/dL, p<0.001), whereas 25-hydroxivitamin D3 level was significant lower (18.29±4.05 ng/mL vs 39.57±7.50 ng/mL, p<0.001). Osteocalcin, marker of osteoblastic activity, was significantly higher in SSc than in control subjects (15.23±3.76 pg/mL vs 6.32±2.01 pg/mL, p<0.001). Also, deoxy-pyridinoline, marker of osteoclast activity, was significantly higher in SSc patients than in the control group (23.13±2.64 pmol/µmol of urinary creatinine vs 19.32±3.31 pmol/µmol of urinary creatinine, p<0.001) (Table 2). In SSc patients 25-hydroxivitamin D3 serum levels significantly correlated (p<0.05 or less) with parathyroid hormone serum levels (r = −0.74, p<0.001) (Fig. 1), BMD measurement in lumbar spine, femoral neck and total femur (r = 0.48, p<0.05; r = 0.52, p<0.01; r = 0.50, p<0.05), Stiffness Index (r = 0.59, p<0.05) and markers of bone turnover, osteocalcin (r = −0.50, p<0,05) and deoxy-pyridinoline (r = −0.56, p<0.05); in SSc group the duration of disease and the inflammatory indices C-reactive protein (CRP) and erythrocyte sedimentation rate (ESR) are not associated with worse outcomes such as lower bone mineral density, fragility fractures, lower serum vitamin D3 levels and higher bone turnover markers and serum parathyroid hormone; No other significant correlation was observed in control group. Lateral spine X-ray documented single or multiple vertebral fractures according to Genant’s criteria in 13 patients (24%), wherease only one subject had a single mild vertebral fracture in control group (1,8%) (Table 2). Eleven patients had a single grade-1 fracture, one patients had a two grade-3 fracture and one patient had a two fractures (one grade-1 and one grade-3). In SSc group the previous use of corticosteroid did not differ significantly between SSc postmenopausal women with or without osteoporosis and the cumulative dose did not correlate with lumbar spine and hip BMD.

**Table 1:** Characteristics of patients in two groups.^a^

Characteristics	SSc (n = 54)	Controls (n = 54)	P
Age (years)	54.43±1.73	54.66±2.87	0.61
BMI (Kg/m^2^)	25.17±4.69	24.22±4.04	0.26
Menopausal Age (years)	51.20±1.74	52.08±1.93	0.43
Pattern of scleroderma, limited/diffuse (%)	34/20 (63/37)	–	N/A
Duration of disease (aa)	5±2	–	N/A
Active disease (Valentini’s activity score ≥3), n(%)	9 (16%)	–	N/A
Severe disease (Medsger’s severity score >1), n(%)	0 (0%)	–	N/A
Smoking status (%)			
Current	14	15	0.88
Former	18	15	0.67
Never	68	70	0.82
Alcohol Drink (ml/week)	600±50	600±50	1.00
*Dietary variables*			
Calcium intake (mg/day)	509±130.65	506±128.88	0.90
Food energy (Kcal/day)	1300±220	1250±150	0.15
Physical activity (%)			
Low	64	60	0.66
Moderate	31	33	0.82
High	5	7	0.66
Supplementation with calcium, n	0	0	N/A
Supplementation with vitamin D, n	0	0	N/A
Weight loss from maximum >5%, n (%)	19 (35)	17 (31)	0.78
Sunlight exposure (%)			
<5 h/week	56	60	0.67
>5 h/week	43	37	0.52
>10 h/week	1	3	0.45
Family history of osteoporosis and/or fractures, n (%)	11 (20%)	10 (18%)	0.79

BMI: body mass index; ^a^results are expressed as means ± S.D. and percentage.

N/A not available.

**Table 2:** Bone mineral density results and laboratory finding in women with SSc and control group.^a^

Parameter	SSc (n = 54)	Controls (n = 54)
Vertebral fractures, n (%)	13 (24)*	1 (1,8)
Lumbar spine		
Bone mineral density (g/cm^2^)	0.78±0.08*	0.88±0.07
T-score (S.D.)	−2.20±0.30*	−1.20±0.30
Femoral neck (S.D.)		
Bone mineral density (g/cm^2^)	0.56±0.04*	0.72±0.07
T-score (S.D.)	−2.60±0.20*	−1.30±0.30
Total Femur (S.D.)		
Bone mineral density (g/cm^2^)	0.57±0.04*	0.71±0.06
T-score (S.D.)	−2.50±0.30*	−1.40±0.30
Stiffness index (SI) (%)	80.10±5.10*	94.80±6.10
Osteocalcin (pg/mL)	15.23±3.76*	6.32±2.01
Deoxypyridinoline (pmol/µmol of urinary creatinine)	23.13±2.64*	19.32±3.31
Parathyroid hormone level (pg/dL)	72.26±11.23*	39.64±11.90
25-hydroxivitamin D3 (ng/mL)	18.29±4.05*	39.57±7.50
Calcium (mg/dL)	9.54±0.48°	9.42±0.63
Phophate (mg/dL)	4.05±0.58°	3.95±0.43
Vitamin A (µg/dL)	59.54±14.48°	62.42±12.63
Creatinine (mg/dL)	0.94±0.18°	0.96±0.13

^a^Data are expressed as means ± S.D.

°P>0.05.

* P<0.001.

## Discussion

We evaluated in a controlled study the prevalence of osteoporosis and vertebral fractures in a postmenopausal patients with systemic sclerosis compared to healthy control matched for age, body mass index, menopausal age and smoking habits. This is the first case-control study of postmenopausal women with SSc to document that they have more prevalent osteoporosis and vertebral fractures than a matched control group. The prevalence of osteoporosis in women affected by SSc was 22%, significantly increased compared to a healthy control group (8%), even if the two groups of postmenopausal women had the same risk factors for osteoporosis (age, menopausal age, BMI, family history for osteoporosis, cigarette smoking, alcohol). This our data are consistent with the current literature [24], [25]. As in our subset, several authors reported no differences in osteoporosis between patients with the limited or diffuse cutaneous subset [26]. In addition did not find an earlier age of menopause in our ssc cohort as compared to a previous study [4]. The analysis performed which no found statistical association between BMD value and previous use of steroid, these finding are consistent with the some authors [26], [27], and the inflammatory indices C-reactive protein (CRP) and erythrocyte sedimentation rate (ESR) is not associated with BMD value, ultrasound parameter and bone turnover markers. These results can be explained by the cross-sectional design of this study and by the few patients who had increased values of CRP and ESR. In our study, careful matching for age and other risk factor for low bone mineral density is an essential factor to interpret the results. Thus, we have matched these factors in order to minimize the influence of this variables. Therefore systemic sclerosis, as it was recently reported by Yuen SY et al. [28], should be considered as an important risk factor for osteoporosis. Lateral spine X-ray documented single or multiple vertebral fractures according to Genant’s criteria in 13 patients (24%), wherease only one subject had a single mild vertebral fracture in control group (1,8%). It is noteworthy that not all patients with fracture had a osteoporosis and vice versa, so these data suggest that qualitative parameters are implicated in fracture risk. Bone ultrasound parameters can measure different physical properties of bone and provide complementary information about fracture risk beyond BMD [29]. In vitro experiences have suggested that QUS may give information not only on bone density but also on architecture and elasticity [30]. In our patients the impairment of stiffness index measured at the heel also provided an additional indication for the presence of a qualitative alteration in the trabecular microarchitecture. Our data showed low hydroxivitamin D levels in SSc patients compared to control subjects. Many studies reported significant association between decreased serum 25-hydroxyvitamin D3 and rheumatic diseases [7]–[14]. Recently Caramaschi P et al showed that low levels of vitamin D are very frequent in patients affected by SSc and patients with vitamin D deficiency showed more severe disease in comparison with patients with vitamin D insufficiency only [17]. Reduced sun exposure, as well intestinal involvement, and renal insufficiency are the main factors which regulate vitamin D3 levels and its status in each subject [28]. The average self-reported sun exposure was similar in both groups and the intestinal involvement and renal insufficiency are not likely the cause of the low vitamin D level; in fact, all our patients showed normal value of prothrombin time and normal value of blood urea nitrogen and creatinine levels which allows to exclude an overt malabsorbiment of liposoluble vitamins and renal insufficiency. These findings can lead us to hypothesize that a skin thickening with capillary damage can result in a reduced drawing of previtamin d3 synthesized from 7-dehydrocholesterol by UV.B radiation in the epidermis or more likely a role for vitamin d deficiency in the pathophysiology of this disease, as suggested for other autoimmune disorders [6]. A comparison of plasma parathyroid hormone levels between our patients and control subjects has shown higher parathyroid hormone levels in the former group. Plasma parathyroid hormone levels showed an inverse correlation with vitamin D3 levels, already reported in other papers [31], and can thus be interpreted as a response correlated with the lower levels of vitamin D3 observed in our patients affected by scleroderma. Urinary levels of deoxy-pyridinoline, marker of osteoclast activity, were higher in our patients compared to control subjects, and serum levels of osteocalcin, marker of osteoblast activity, followed a similar trend. Thus, the bone deficit found in these newly diagnosed patients seems likely to be a result of a recent acceleration of bone turnover It is reasonable to believe that all of which could contribute to an increased factor risk for osteoporosis and fragility fracture. Cauley et al showed a linked between low 25(OH)D levels and risk fracture in white women [32]. Our study has limitations that must be considered in the interpretation of our finding. Firstly, our study including a small sample size. Another limitation was not to compare the population of scleroderma with another chronic disease to better understand the impact and specificity of scleroderma on bone loss and fragility fractures. Further studies on the effects of vitamin D supplementation on BMD in these patients could confirm the impact of vitamin D deficiency on bone mass in systemic sclerosis disease. In conclusion, our data showed, for the first time, higher prevalence on vertebral fractures in postmenopausal women with systemic sclerosis and that low vitamin D3 level seem may contribute to bone loss in this patient.
